# Microparticles as autoantigens in human and murine lupus

**DOI:** 10.1186/ar3970

**Published:** 2012-09-27

**Authors:** A Ullal, DS Pisetsky

**Affiliations:** 1 Duke University Medical Center, Durham, NC, USA; 2 Medical Research Service, Durham Veterans Administration Medical Center, Durham, NC, USA

## 

Systemic lupus erythematosus is a systemic inflammatory disease characterized by anti-DNA production in association with immune complex deposition. These complexes can deposit in the tissue to incite inflammation as well as stimulate cytokine production by plasmacytoid dendritic cells. While the properties of anti-DNA antibodies have been extensively characterized, little is known about the nature of the DNA in the immune complexes although its origin is generally considered to be cell death. Among sources of extracellular DNA, microparticles (MPs) are small membrane-bound vesicles released from activated and dying cells by a blebbing process. As shown by flow cytometry, these particles display DNA as well as other nuclear molecules in an antigenically accessible form as indicated by the binding of monoclonal anti-DNA antibodies as well as plasma from patients with lupus. Furthermore, plasma from patients with lupus contains increased number of particles with bound IgG, suggesting that MPs can form immune complexes found in lupus. To assess whether MPs play a similar role in murine lupus, we used flow cytometry to measure the presence of MPs with bound IgG in the blood of MRL-*lpr/lpr *and NZB/W mice; in addition, we tested the binding of plasma of these mice to MPs from apoptotic cells. The results of these studies showed important differences in the serological findings of the two strains as reflected by the much higher numbers of MPs with bound IgG in the blood of MRL-*lpr/lpr *compared with NZB/W mice. These studies also showed that antibodies from MRL-*lpr/lpr *mice bound much better to MPs from apoptotic cells than those from NZB/W mice. Since particles in NZB/W blood bound to monoclonal anti-nuclear antibodies as well as MRL-*lpr/lpr* plasma, these findings indicate antigenic activity despite the lack of particle IgG complexes in NZB/W blood. Together, these studies indicate important differences in the serological features of the two strains as reflected by the capacity of antibodies to bind to MPs. These differences may impact on the process of immune complex formation and its consequences as well as the respective role of anti-DNA and other autoantibodies in nephritis in NZB/W mice.

